# *Antarctotrechus balli* sp. n. (Carabidae, Trechini): the first ground beetle from Antarctica

**DOI:** 10.3897/zookeys.635.10535

**Published:** 2016-11-23

**Authors:** Allan C Ashworth, Terry L. Erwin

**Affiliations:** 1 Quaternary Entomology Lab, Department of Geosciences, North Dakota State University, Fargo, North Dakota 58108, USA; 2 Hyper-diversity Group, Department of Entomology, MRC-187, National Museum of Natural History, Smithsonian Institution, Washington, P.O. Box 37012, DC 20013-7012, USA

**Keywords:** Trechini, Carabidae, Antarctica, Miocene

## Abstract

Fossil elytra of a small trechine carabid are reported from the Oliver Bluffs on the Beardmore Glacier at lat. 85°S. They were compared with counterparts from the extant genera *Trechisibus*, *Tasmanorites*, *Oxytrechus* and *Pseudocnides*. The fossils share some characters but are sufficiently different to be described as a new genus and species. We named the new species *Antarctotrechus balli* in honour of George E. Ball who made major contributions to the study of carabids through his own research and the training of students while at the University of Alberta, Edmonton, Alberta, Canada. The closest extant relatives to the extinct *Antarctotrechus balli* are species of *Trechisibus*, which inhabit South America, the Falkland Islands and South Georgia, and *Tasmanorites*, which inhabit Tasmania, Australia. Plant fossils associated with *Antarctotrechus balli* included *Nothofagus* (southern beech), *Ranunculus* (buttercup), moss mats and cushion plants that were part of a tundra biome. Collectively, the stratigraphic relationships and the growth characteristics of the fossil plants indicate that *Antarctotrechus balli* inhabited the sparsely-vegetated banks of a stream that was part of an outwash plain at the head of a fjord in the Transantarctic Mountains. Other insects represented by fossils in the tundra biome include a listroderine weevil and a cyclorrhaphan fly. The age of the fossils, based on comparison of associated pollen with ^40^Ar/^39^Ar dated pollen assemblages from the McMurdo Dry Valleys, is probably Early to Mid-Miocene in the range 14–20 Ma. The tundra biome, including *Antarctotrechus balli*, became extinct in the interior of Antarctica about 14 Ma and on the margins of the continent by 10–13 Ma. *Antarctotrechus balli* confirms that trechines were once widely distributed in Gondwana. For *Antarctotrechus balli* and other elements of the tundra biome it appears they continued to inhabit a warmer Antarctica for many millions of years after rifting of Tasmania (45 Ma) and southern South America (31 Ma).

## Introduction

Insects are least well-represented in Antarctica than anywhere else on Earth. The living fauna consists of three species of flightless chironomid midges. Two of these are considered native and one is adventive. Molecular analysis confirms that *Belgica antarctica* and *Eretmoptera murphyi* Schaeffer are closely related in the subfamily Orthocladiinae and represent an ancient Antarctic lineage. *Parochlus steinenii* (Gerke), the third species, is in the subfamily Podonominae and more closely related to an older lineage from Tierra del Fuego and South Georgia (Allegruchi et al. 2006). Both of the native species occur on the west side of the Antarctic Peninsula and offshore islands, and also South Shetland, South Orkney and the South Sandwich Islands (Convey and Block 1996). Only one of them, *Belgica antarctica* Jacobs, 1900, is truly restricted to the Antarctica Peninsula and subantarctic islands. The lack of available moisture, low temperatures and vegetation are considered limiting factors. The furthest south *Belgica antarctica* is known from is 68°S (Convey and Block 1996).

The fossil record for insects in Antarctica is equally poor. Older records are reviewed by Carpenter (1969). Impressions of wings and distorted bodies in late Paleozoic to middle Mesozoic-aged carbonaceous shales have proved difficult to assign to anything other than the broadest categories e.g. Odonata, Homoptera and Coleoptera. Newer records for insects are from Neogene-aged deposits in the Transantarctic Mountains. They differ from the older insects in that they are pieces of exoskeletal elements, not impressions. A fragment of an elytron, possibly a curculionid, was reported from 14 million year old deposits in the Olympus Range in the McMurdo Dry Valleys (Lewis et al. 2008). Exceptionally well-preserved beetle fragments, including a pronotum covered in fine setae, were reported from a soil profile in the New Mountain area, also in the McMurdo Dry Valleys (Mahaney et al. 2012). The specimens were said to be of Miocene age but because of the preservation of the fine setae we do not accept that the specimens are fossil. Most probably they represent parts of a modern beetle that had contaminated the sample in storage in North America before analysis.

The fossil trechine we report is from the same stratigraphic horizon as a head and a leg of a listroderine weevil (Ashworth and Kuschel 2003) and the posterior segments of a puparium of a cyclorraphan fly (Ashworth and Thompson 2003)

## Location and stratigraphy of the Meyer Desert Formation

**Figure 1.**
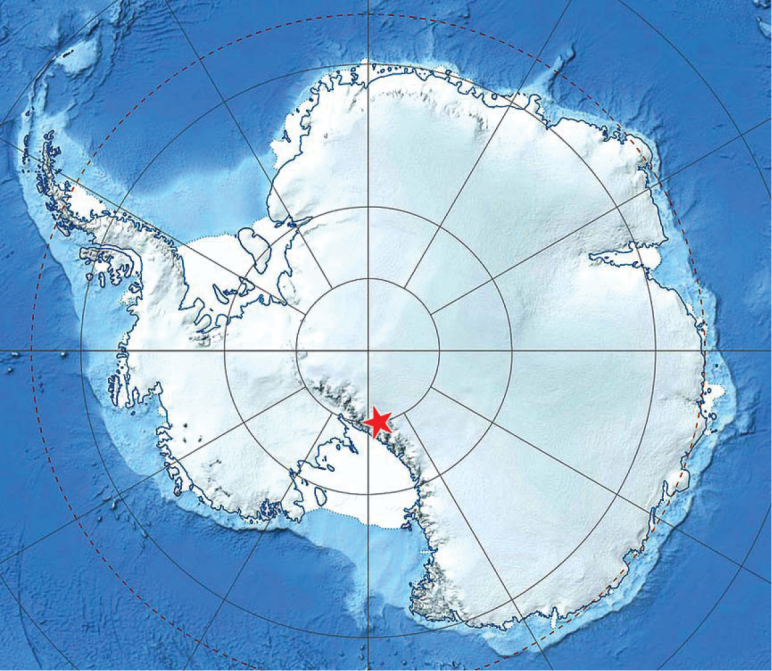
The type locality for *Antarctotrechus balli* sp. n. is shown by the red star. Image map is a modified MODIS Mosaic of Antarctica from National Snow and Ice Data Center, http://nsidc.org/data/moa/

**Figure 2.**
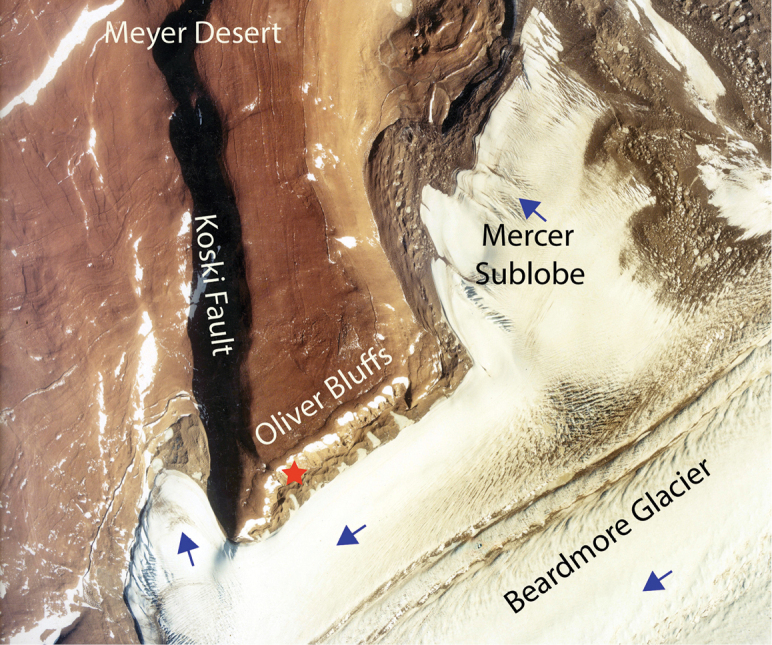
An aerial view of the Oliver Bluffs on the Beardmore Glacier. Ice flow directions are shown by the blue arrows. The Meyer Desert Formation is downthrown to the north along the Koski Fault. The type locality for *Antarctotrechus balli* sp. n. is shown by a red star. The aerial image # TMA-2738-4 is from the collections of the The United States Antarctic Resource Center (USARC), USGS, Reston, VA.

**Figure 3.**
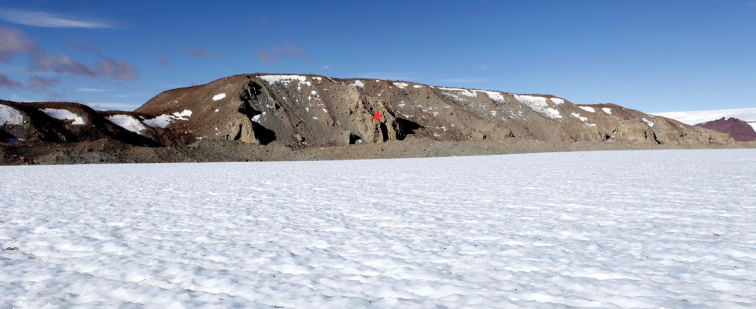
Ancient glacial deposits of the Meyer Desert Formation exposed in the Oliver Bluffs on the flanks of the Beardmore Glacier. The deposits are downthrown along the Koski fault which is marked by the prominent escarpment towards the north end of the bluffs (left on image). The type locality for *Antarctotrechus balli* sp. n. is at the north end of the bluffs marked by a red star.

**Figure 4.**
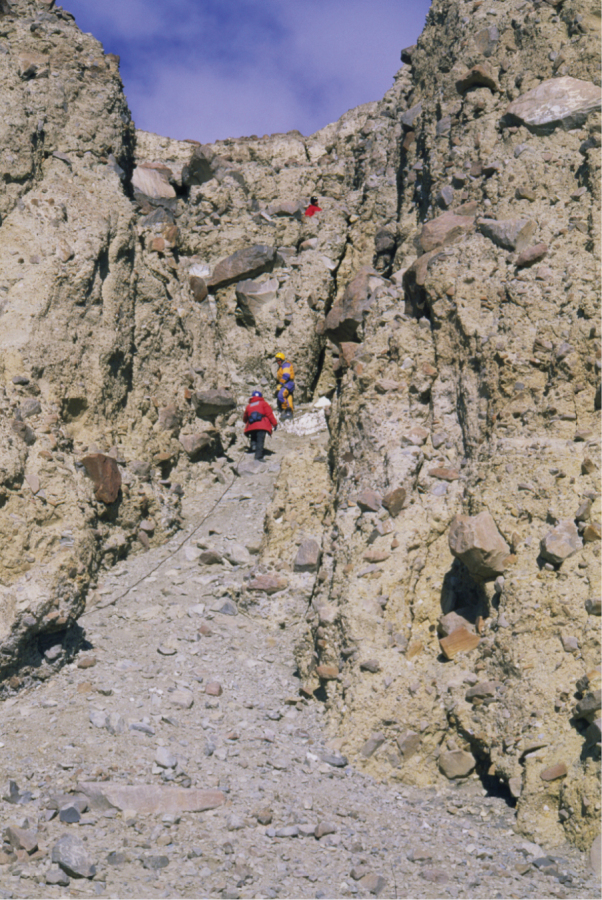
The type locality for *Antarctotrechus balli* sp. n. is a siltstone lens within a sequence of lodgement tills exposed in the gulley wall to the left of the person highest in the gully.

The fossils come from the Oliver Bluffs on the Beardmore Glacier which is a major outlet glacier of the East Antarctic Ice Sheet. The site is within the Transantarctic Mountains about 550 km from the South Pole (Figure 1). As uplift occurred on the flanks of the Ross Sea rift, the Beardmore Glacier became progressively entrenched leaving older glacial deposits on the flanks (Figures 2–4). The present elevation of the Oliver Bluffs is 1731m above msl. However, based on a report of agglutinated foraminifera from the basal deposits at the Oliver Bluffs (Webb et al. 1994), it is probable that the deposits in which *Antarctotrechus balli* and other fossils were preserved accumulated near sea-level on the margins of a fjord. The older glaciogene deposits of the Beardmore Glacier region are referred to as the Mt. Mills, Meyer Desert and Cloudmaker Formations of the Sirius Group, formerly Sirius Formation (McKelvey et al. 1991; Webb et al. 1996). The Meyer Desert Formation is the youngest of these and the only one known to contain *in situ* fossils from terrestrial and freshwater habitats. The trechine fossils come from a siltstone lens located about 20 m above the base of a bluff located at 85.117222°S, 166.657500°E (Figures 2–4).

The lens is interbedded within large boulder diamictites and is part of a horizon containing outwash sandstones and conglomerates, laminated proglacial lake deposits, debris flows, peat beds and shallow lacustrine mudstones. The siltstone lens formed from the infilling of a stream channel. Insect and plant parts were washed or blown into the stream channel which eventually became sediment-choked and finally buried by lodgement till during the next glacial advance down the valley. The Oliver Bluffs stratigraphy supports the interpretation that the stream was part of a broad outwash plain that extended between the glacier margin and the fjord (Ashworth and Cantrill 2004).

### Paleoecology

The Oliver Bluffs on the Beardmore Glacier is one of the most important Neogene terrestrial paleontological sites in Antarctica. The possibility that fossils might be found there was first noted by John Mercer. He recognized that the wet-based glacial sediments exposed in the bluffs could only have been deposited at the time when the climate was warmer and wetter than it is now (Mercer 1972). On a later expedition, fossil wood was discovered (Webb and Harwood 1987) and assigned to *Nothofagus* (Carlquist, 1987). This was followed by the discovery of a mat of *Nothofagus* leaves, one-leaf thick. The leaves have similar size and shape to the extant sub-alpine *Nothofagus gunnii* from Tasmania. They have a different veination pattern, however, which led to them being described as an extinct species, *Nothofagus beardmorensis* (Hill et al., 1996). An analysis of tree rings of the abundant twig-sized *Nothofagus* wood indicated prostate shrubs rather than trees (Francis and Hill 1996). Pollen from the site is represented by a single species of *Nothofagus*, *Nothofagus lachlaniae* (Askin and Markgraf 1986). Other plant fossils from the site, notably abundant achenes of *Ranunculus* and cushion plants of both moss and an angiosperm species, indicate a patchy shrub tundra (Ashworth and Cantrill 2004). The habitat for the new species of trechine was the sparsely vegetated sand and gravel banks of a meltwater-fed stream.

### Age of the deposits

Based on the occurrence of marine diatoms within the Sirius Group deposits, their age was reported as Pliocene c. 3 Ma (Webb et al. 1994). This age estimate, however, is controversial and likely too young. Several studies suggest that the diatoms were deposited onto older glacial deposits by wind from Pliocene marine deposits located on the margins of the continent (Scherer et al. 2015). Two lines of evidence from the Oliver Bluffs, one geomorphological and the other palynological, also support an older age for the deposits.

The Meyer Desert Formation at the northern end of the Oliver Bluffs is off-set by splay faults associated with the Koski Fault (Figure 2). Surface exposure ages of Beardmore moraine boulders overlying the Meyer Desert Formation, and offset by faults, are between 1.9-5.8 Ma (Ackert and Kurz 2004). The estimate assumed no erosion and constant elevation leading the authors of the study to conclude that the Meyer Desert Formation had to be much older than c.3 Ma.

Pollen from the Meyer Desert Formation most closely matches an assemblage from near Mount Boreas in the McMurdo Dry Valleys dated by ^39^Ar/^40^Ar from a volcanic ash to be of mid-Miocene age (14.07 ± 0.05 Ma., Lewis et al. 2008). Also, fossiliferous Neogene deposits in the region of the Friis Hills in the McMurdo Dry Valleys are early to mid-Miocene based on ^39^Ar/^40^Ar dating (Lewis and Ashworth 2016). We conclude that the best age estimate for the trechine fossils is mid-Miocene. Certainly it is possible that the retreat of glaciers and colonization by a tundra biota correlates with the mid-Miocene climatic optimum, a well-known globally warm climatic event (Flower and Kennett 1995, Warny et al. 2009, Feakins et al. 2012).

### Fossil preparation

The fossiliferous siltstone is calcite-cemented which disaggregated after soaking in water. The grains were further separated by washing in a jet of water and those that remained on a 300µ mesh were then examined under a binocular microscope (20×). Fossils picked from the sediment matrix in addition to the trechine elytra included twigs of *Nothofagus* (southern beech) wood, moss stems, seeds of *Ranunculus* (buttercups) (Ashworth and Cantrill 2004).

## Description

### Antarctotrechus

Taxon classificationAnimaliaColeopteraCarabidae

Ashworth & Erwin gen. n.

http://zoobank.org/698F1D95-6514-4AED-8E6A-F29EBD6C6014

#### Type species.

*Antarctotrechus balli* Ashworth & Erwin, sp. n.

### Antarctotrechus balli

Taxon classificationAnimaliaColeopteraCarabidae

Ashworth & Erwin sp. n.

http://zoobank.org/B5A220C2-B707-489D-ACCD-03980BD2A231

#### Holotype

(sex unknown), a right elytron. **Antarctica**, Oliver Bluffs, Beardmore Glacier region, Meyer Desert Formation, 85.117222°S, 166.657500°E, (Allan Ashworth 2003) (NMNH: ADP147741).

#### Derivation of genus name.

*Antarctotrechus* refers to its relationship with the Trechini and the place where the specimens were found.

#### Derivation of specific epithet.

The epithet, *balli*, is a Latinized eponym based on the family name of George E. Ball, Carabidologist, and academic leader of a host of younger carabidologists, in celebration of his 90^th^ birthday, September 26, 2016.

#### Proposed english vernacular name.

Ball’s Antarctic Tundra Beetle.

#### Diagnosis.

**Figure 5.**
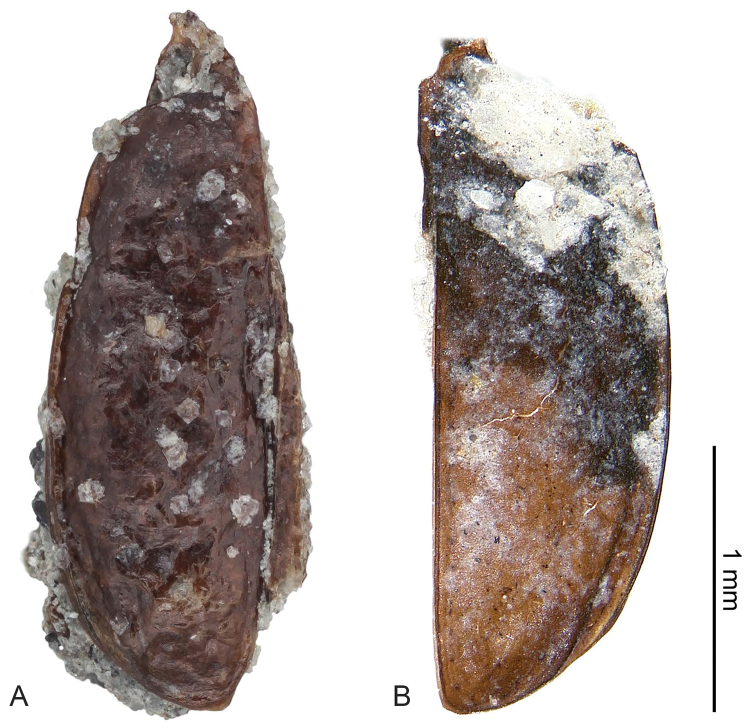
Fossils of the left and right elytra of *Antarctotrechus balli* sp. n. **A** The left elytron, 2.36 mm in length, is designated as the paratype (USNM: ADP147732). A small part of the right elytron of the same individual is attached along the suture near the apex. The siltstone matrix is visible along the outer margin near the apex. The small rhomb-shaped crystals on the surface are authigenic calcite **B** The right elytron is designated as the holotype. (NMNH: ADP147741). The elytron is 2.40 mm in length. The base is deformed by a crack and concealed by sediment grains. A small part of the left elytron of the same individual is still attached along the suture near the base.

**Figure 6.**
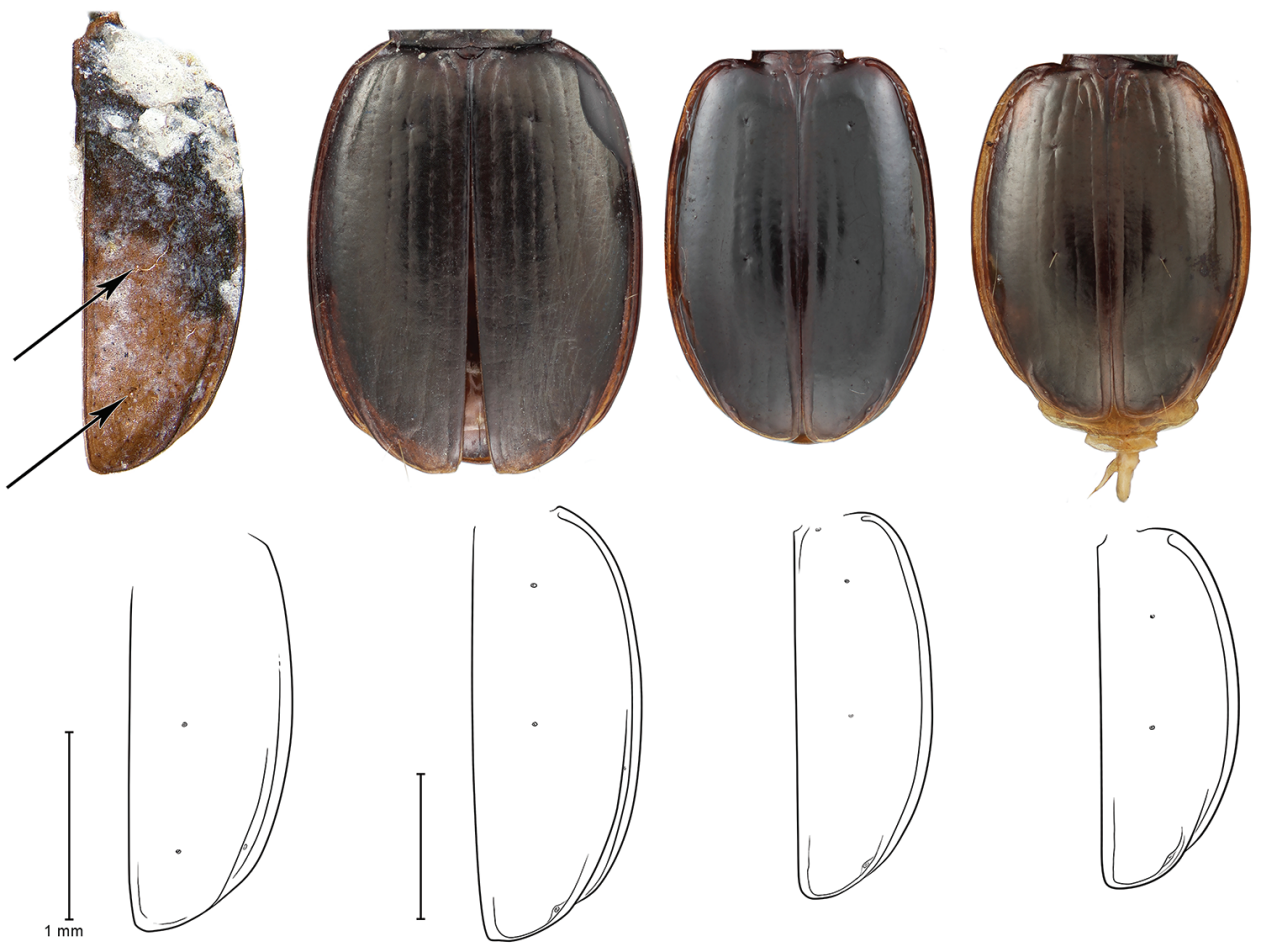
Fossil of right elytron of *Antarctotrechus balli* sp. n. and elytra of three species of Neaustral *Trechisibus* spp. with line drawings of each showing 3^rd^ interval setigerous pores and recurrent grooves of the latter.

**Figure 7.**
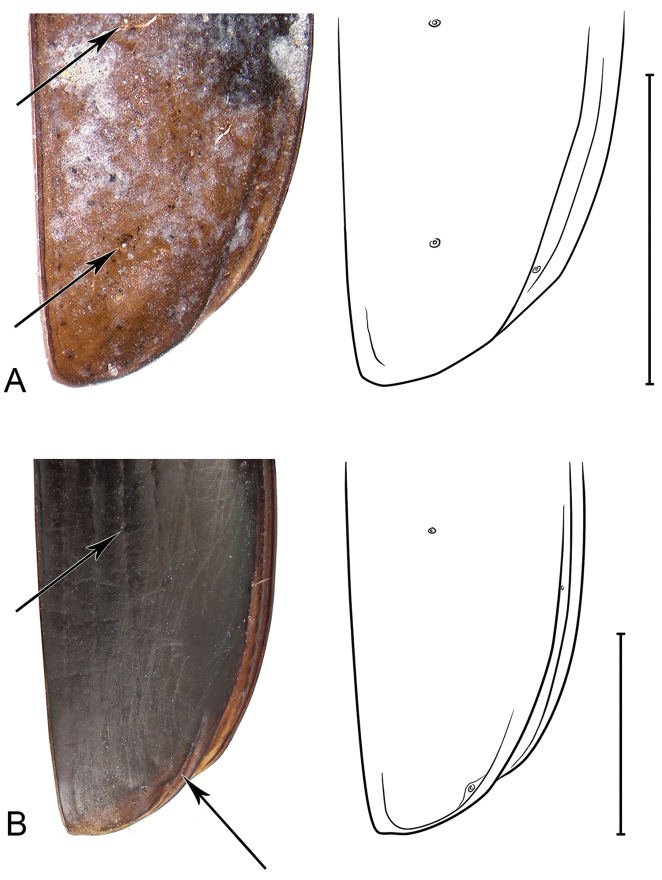
Comparison of the apices of the fossil right elytron *Antarctotrechus balli* sp. n. and a modern *Trechisibus* sp. with line drawings of each showing 3^rd^ interval setigerous pores and recurrent grooves of the latter. An isodiametric microornament is partially visible on the fossil.

Only the left and right elytra known (Figure 5). They are from two individuals. Form as in alate Neaustral trechines in the genus *Trechisibus* Motschulsky 1862. Lack of recurrent groove as in Andean and Pampas trechines in the genera *Oxytrechus* Jeannel 1927 and *Pseudocnides* Jeannel 1927, respectively. Placement of mid-discal elytral setiferous pore as in *Trechisibus* and other trechines, Placement of preapical in third interneur unique (Figures 6–7). Absence of apical elytral setiferous pore in third interneur unique.

#### Description.

(Figures 5–7). *Size*: Elytron length and width within range of *Trechisibus* species, Length 2.36 mm, W = 0.85 mm.

*Color*: Typically trechine brown.

*Luster*: Unknown due to deposition and lithification processes over ~ 20 -14 Ma.

*Microsculpture*: Apparently isodiametric.

*Head*: Unknown.

*Prothorax*: Unknown.

*Pterothorax*: Shape of humerus (compare Figure 5) not sloped, indicating the adult was possibly fully winged.

*Legs*: Unknown.

*Abdomen*: Unknown.

*Male genitalia*: Unknown.

*Female genitalia*: Unknown.

#### Dispersal potential.

If these beetles were macropterous (see above), they were likely capable of flight. *Trechisibus* adults are moderately swift and agile runners, so likely were adults of *Antarctotrechus balli*. All known species of *Tasmanorites* are brachypterous.

#### Other specimens examined.

A left elytron also from the type locality is designated as the paratype. This specimen is reposited in the collections of the Smithsonian Institution National Museum of Natural History (NMNH:ADP147732).

### Systematics and biogeographic significance

Our search for relationships of the fossil species focused on four genera of southern trechines: *Trechisibus* Motschulsky 1862, *Tasmanorites* Jeannel, 1927, *Oxytrechus* Jeannel, 1927, and *Pseudocnides* Jeannel, 1927. *Oxytrechus* and *Pseudocnides* share with the fossil a unique feature in trechines, i.e., lack of a recurrent groove, however, neither is associated with *Nothofagus* (southern beech) forests, rather they are for the most part montane and lowland grassland species (La Puna and La Pampa). In addition, the latter two genera are not truly Neaustral, rather Andean or Pampean.

**Figure 8.**
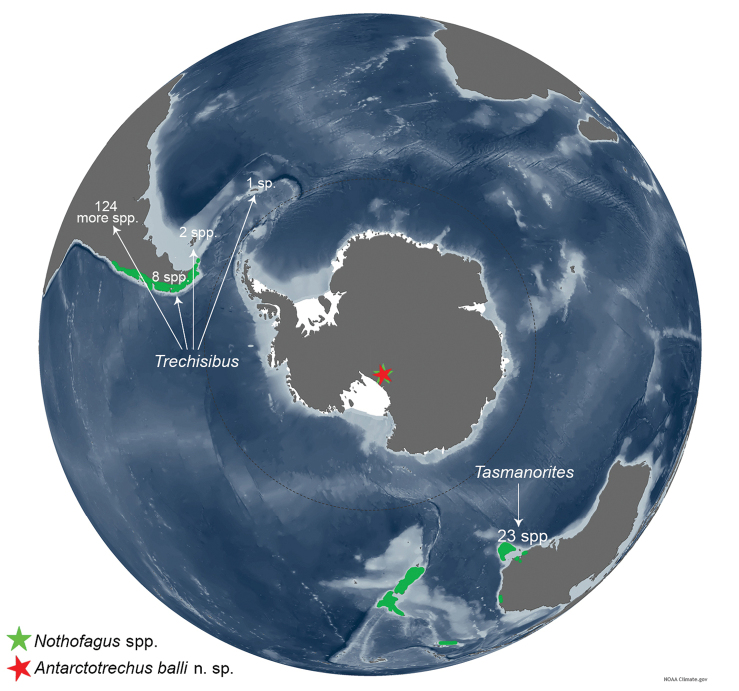
The distribution of extant species of *Trechisibus*, *Tasmanorites* and *Nothofagus* and the fossil occurrence of *Antarctotrechus balli* sp. n. and *Nothofagus beardmorensis* on the Beardmore Glacier shown by red and green stars: information for *Trechisibus* species (Allegro et al. 2008) and *Tasmanorites* (Eberhard S, Giachino, PM, 2011). Base image: NOAA Climate.gov https://www.climate.gov/news-features/understanding-climate/polar-opposites-arctic-and-antarctic

With the exception of the lack of a recurrent groove, the fossils are similar in size and shape and in the placement of mid-discal elytral setiferous pore to *Trechisibus* and *Tasmanorites*. These genera are very closely related (David Maddison, Oregon State University, pers. comm., based on molecular studies). Trechisibus is abundant in the fauna of southernmost South America extending northward to the Andes in Peru and Ecuador. *Tasmanorites* occurs in Tasmania but not on the mainland of Australia or New Zealand. *Trechisibus* and *Tasmanorites* have a circum-Antarctic relationship and both are associated with *Nothofagus* forests, as was the new genus and species *Antarctotrechus balli* (Figure 8).

The conflicting signals both in anatomical attributes and biogeography, and in ecological setting as well, leave open the question of relationships, thus giving us no alternative but to flag the species represented by fossil evidence through erection of new genus status, hence drawing attention to it and the need for further paleontological studies in Antarctica.

The type locality of *Antarctotrechus balli* is midway between Tasmania and southernmost Patagonia (4600 vs 4300 km) suggesting that *Trechisibus*-*Tasmanorites*-like clades occupied a vast area of the southern land masses prior to ~14 Ma ago (Figure 8). There is no known *Trechisibus*-*Tasmanorites*- like clade in New Zealand where the southernmost part is only 3 degrees further north than the southernmost part of Tasmania. The absence of the clade in New Zealand adds fuel to the controversial hypothesis that the original Gondwana biota of New Zealand was drowned during marine transgression in the Oligocene and/or the early Miocene (Mildenhall et al. 2014).

Populations of *Antarctotrechus balli* had probably become extinct in the Beardmore Glacier region (lat. 85°S) by ~14 Ma ago, or earlier. Further north in the Olympus Range (lat. 77°S), in the McMurdo Dry Valleys, major climate change at ~14 Ma ago resulted in the extinction of the tundra biota (Lewis et al. 2008). The warmth and moisture needed to support patches of tundra with *Nothofagus*, and populations of *Antarctotrechus balli*, may have persisted on the margins of the continent (lat. 65°S) until 10-13 Ma ago (Anderson et al. 2011, Wei et al. 2013). Eventually, however, the climate became too cold and too dry to support anything but extremophiles.

## Supplementary Material

XML Treatment for Antarctotrechus

XML Treatment for Antarctotrechus balli
